# Utilizing a health information exchange to facilitate COVID-19 VA primary care follow-up for Veterans diagnosed in the community

**DOI:** 10.1093/jamiaopen/ooab020

**Published:** 2021-03-16

**Authors:** Rachel L Sherman, Kimberly M Judon, Nicholas S Koufacos, Vivian M Guerrero Aquino, Shaniqua M Raphael, Judith T Hollander, Kenneth S Boockvar

**Affiliations:** Department of Veterans Affairs, James J. Peters Medical Center, Bronx, New York, USA

**Keywords:** health information exchange, COVID-19, coronavirus, health information interoperability, veterans health

## Abstract

The use of alerts from the Bronx RHIO, a health information exchange (HIE) to identify James J. Peters VAMC patients diagnosed with COVID-19 in the community was described to facilitate COVID-19 VA primary care follow-up. COVID-19 hospitalization and testing alerts were delivered on a Bronx RHIO facility report. VA COVID-19 follow-up care by telephone and video was guided by local COVID-19 clinical pathways, electronic health record (EHR) templates, and tracking through a database. VA received 180 RHIO alerts for 111 unique patients, and 88 had positive non-VA testing from March to June 2020. 41% of the 88 had non-VA admissions and 23% died. 63% received VA primary care follow-up of COVID-19 symptoms documented by custom EHR templates. The HIE identified 11% of the facility COVID-19 patients. HIE alerts can be used to identify facility COVID-19 patients diagnosed in the community and facilitate follow-up by their VA primary care teams.

LAY SUMMARY

Veterans receive care in both VA and community settings including during the COVID-19 pandemic. At the James J. Peters VA in Bronx, Veterans with COVID-19 symptoms, some previously hospitalized, receive telephone, less often video, follow-up care from their primary care teams until resolution of symptoms. The follow-up includessymptom assessment and management, triage to a higher level of care if needed, mental health care, and infection control advice for patients and their caregivers. The hospital wished to identify its patients diagnosed with COVID-19 outside of the VA and extend the same follow-up care by their familiar primary care teams. This study describes how the facility used their local health information exchange (HIE), an electronic method to share health information between facilities, to identify their patients diagnosed outside the VA, and provide them follow-up of COVID-19 symptoms during the New York pandemic surge from March to the end of June 2020. Bronx RHIO HIE alerts identified 88 patients with positive non-VA COVID-19 testing. 63% received VA primary care COVID-19 symptom follow-up. 41% were hospitalized at non-VA sites and 23% died. 11% of the total facility patients (813) diagnosed with COVID-19 by the end of June 2020 were from the Bronx RHIO.

## INTRODUCTION

New York City was the epicenter of COVID-19 in the spring of 2020. In New York City, between March and the end June of 2020, there were over 200,000 cases of COVID-19 and over 50,000 hospitalizations. There were almost 50,000 cases in the Bronx where the James J Peters VA Medical Center (JJP VAMC) is located.

At the outset of the pandemic, JJP VAMC developed a program with local subject matter experts from infection control, primary care, informatics, and nursing to provide ambulatory assessment, triage, and management of facility patients with COVID-19 symptoms, using clinical pathways. Follow-up care was conducted by nurses and licensed independent practitioners primarily via telephone and secure video through the VA video platform, VA Video Connect. Some clinicians and patients corresponded via secure messaging via the VA’s health portal, My HealtheVet. The COVID-19 ambulatory program content was standardized through locally developed electronic health record (EHR) templates which were updated as national VA COVID-19 templates were created and as public health recommendations evolved.^1–3^ For patients appropriate for ambulatory management of COVID-19 and after emergency department and inpatient discharge, follow-up care included serial symptom assessment and management, pulse oximeter and temperature review when available, mental health assessment, and infection control advice until resolution of symptoms or triage to a higher level of care when required. Family and caregivers of deceased patients also received bereavement support and infection control advice.

During the COVID-19 pandemic, most facility Veterans were tested and treated at the JJP VAMC, but facility patients were also tested, treated, and admitted for COVID-19 at non-VA sites. Knowledge of non-VA COVID-19 test results and treatment was essential to provide the same ambulatory COVID-19 primary care follow-up as patients diagnosed at the facility. However, there was no local or national standard process in place to identify VA patients diagnosed with COVID-19 in the community and to relay it to their VA primary care teams to initiate ambulatory symptom follow-up.

Health information exchange (HIE) is the one method by which VA clinicians obtain healthcare information from local community healthcare systems such as for test results and admissions. Health information exchange is defined as the electronic transfer of clinical, administrative, or other information necessary for the delivery of health care across diverse systems or organizations.^4^^,^^5^

Lenert et al. wrote about the importance of health information exchanges for COVID-19 in the June 2020 JAMIA: “The coronavirus disease 2019 (COVID-19) response imposes unprecedented challenges on our healthcare system. To address these challenges, the health information needed to safely care for a patient needs to flow across provider platforms in a given region without impediment”.^6^

The JJP VAMC is a partner with the Bronx Regional Health Information Organization (Bronx RHIO), a local HIE. VA providers have had access to the Bronx RHIO since 2008^7^ and use it to obtain clinical data including laboratory and radiology studies, emergency department visits, and community hospital admissions. The Bronx RHIO also provides acute event notification to JJP VAMC staff.

## OBJECTIVE

To describe the use of alerts from the Bronx RHIO, a health information exchange, to identify patients enrolled at the JJP VAMC diagnosed with COVID-19 in the community to facilitate COVID-19 follow-up by their VA primary care teams during the New York City pandemic surge from March to June 2020.

## MATERIALS AND METHODS

The setting for the study is the JJP VAMC located in the Bronx, New York. The JJP VAMC is an academic tertiary care VA medical center with comprehensive inpatient services, three community clinics, a nursing home, and a long-term spinal cord unit.

**Figure 1.**
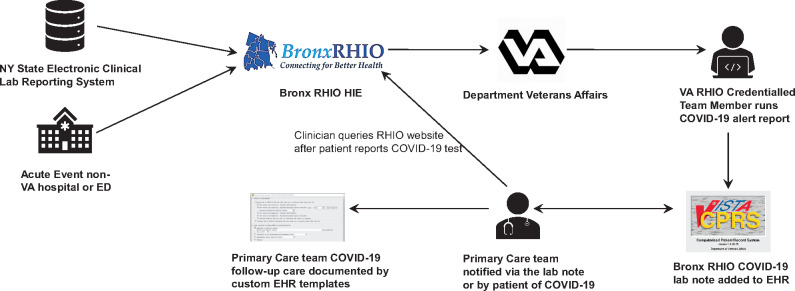
Schematic of the delivery of COVID-19 data from the Bronx RHIO to the VA RHIO credentialled team members and subsequently to VA primary care to provide follow-up of COVID-19 symptoms documented through EHR custom templates.

In this study, alerts of hospitalization events and COVID-19 molecular and serology test results were delivered on a facility patient roster via a Bronx RHIO report accessed by a RHIO-credentialed VA staff member to facilitate downstream ambulatory follow-up by the patient’s VA primary care team. Primary care COVID-19 serial symptom assessment, triage, and management follow-up were documented in custom electronic templates in the VA EHR, VistA. Primary care team members tracked their COVID-19 patients through a custom local primary care COVID-19 database (Figure 1). This study was approved as a quality improvement by the local institutional review board.

VA RHIO-credentialled staff members checked the Bronx RHIO COVID-19 roster daily and entered the non-VA results into the EHR through a template created to document outside COVID-19 molecular and serology results with structured data elements. The primary care team was notified of the test results through the template via a signature request. For some patients, the primary care team directly queried the RHIO and documented outside results on the same template after patients informed them of their outside COVID-19 results.

Primary care staff used clinical pathways to guide their ambulatory follow-up of COVID-19 patients diagnosed at non-VA sites, using the same EHR templates for documentation with structured data elements used for patients diagnosed at the facility. The serial follow-up EHR template included structured symptom assessment and management, triage to a higher level if care for worsening symptoms, mental health assessment, and infection control advice. A discharge from monitoring template documented resolution of symptoms with guidance on release from home isolation. Templates were not used in secure messaging correspondence.

Primary care teams tracked their COVID-19 patients with a custom facility primary care COVID-19 database report. Summary statistics were calculated for the patient cohort using SAS version 9.4 (SAS Institute, Cary, NC).

Due to a New York State executive order, the requirement for a veteran to consent for RHIO access was waived during this time period. Since March 2020, The Bronx RHIO has provided COVID-19 alerts and reporting customized for each member organization including the JJP VAMC.^8^ The Bronx RHIO integrates COVID-19 results from the state-provided Electronic Clinical Lab Reporting System (ECLRS) public health data with member organization provided data for this reporting.^8^

## RESULTS

A total of 180 COVID-19 Bronx RHIO alerts for 111 unique patients were received by RHIO VA-credentialed team members from April 6, 2020 through June 23, 2020 for non-VA testing, after alerting was turned on starting March 14, 2020. Of those patients, 88 (79%) had positive COVID-19 molecular and/or serology testing performed at non-VA sites. During this time, additional 725 JJP VAMC patients were diagnosed with COVID-19 via within-facility laboratory testing. Thus, non-VA outside-system testing contributed 88 of the 813 JJP VAMC patient cases (11%).

Of the 88 patients who had a positive non-VA COVID-19 laboratory test result, 36 (41%) were admitted to hospitals in the community and 12 (14%) died in those facilities. Combined VA and RHIO data revealed a total of 20 (23%) deaths following a positive non-VA COVID-19 laboratory test result.

**Figure 2.**
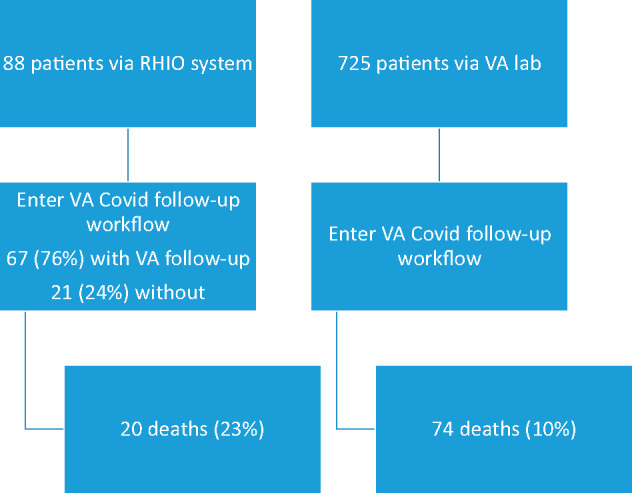
COVID-19 cases at a VA facility with source of case identification (RHIO vs VA lab), mortality, and follow-up.

The VA RHIO team informed primary care of positive COVID-19 testing for 65 (74%) of the 88 patients. For the remaining 23, the primary care team had documented the positive outside testing prior to the alert through other information channels. Thus, the RHIO identified at least 65 COVID-19 patients who would not otherwise have been identified within the VA facility. Overall, 55 (63%) of the 88 patients with outside COVID-19 positive results had VA primary care COVID-19 pathway-guided ambulatory follow-up care documented through custom COVID-19 her-templated notes and additional 12 patients (14%) had follow-up for COVID-19 with any VA clinician in the EHR without template use as per chart review.

## DISCUSSION

This study presents an example of a process to utilize health information exchange alert notification to identify VA patients diagnosed with COVID-19 in the community to facilitate ambulatory telephone and less often video follow-up by their VA primary care team to manage their COVID-19 infection. The primary care COVID-19 symptom assessment and management were documented through custom EHR templates and tracked through a custom primary care COVID-19 data analytics report. The HIE alert process enabled Veterans diagnosed with COVID-19 outside the VA to receive the same serial symptom follow-up by their established primary care teams as those diagnosed at the facility. The process was created early in the pandemic when New York City was overwhelmed by admissions and recommended home management for most patients for COVID-19 symptoms unless severe.^2^

For the COVID-19 pandemic, the laboratory result alerts supplied by the Bronx RHIO provided 11% of the total positive COVID results for the facility as of July 2020. Approximately 63% of facility patients with COVID-19 RHIO alerts subsequently received COVID-19 follow-up care by their primary care teams via custom EHR templates and 76% received follow-up by any VA clinical staff member with or without the templates as per chart review. Possible explanations of lack of documented EHR follow-up COVID-19 care include delayed education on template use for some primary care teams, redeployment of staff to inpatient and emergency areas, follow-up calls without EHR documentation, COVID-19 follow-up via secure messaging in the VA health portal, and the large number of outpatients with COVID-19 symptoms VA primary care staff were tracking during this time period.

Bronx RHIO alerts accounted for approximately 21% of facility-wide COVID-19 deaths by July 2020. Veterans with COVID-19 identified through non-VA testing likely died at a high rate because non-VA sites were only testing severely ill hospitalized patients initially and the JJP VAMC extended testing to outpatients by late March. As such, the Bronx VA RHIO alerts for non-VA testing were for severely ill patients whereas the JJP VAMC test results included inpatients and outpatients. Swab, testing reagent and personal protective equipment shortages early in the pandemic influenced local facility and public health policy on testing.^2^

A major limitation of this study’s COVID-19 alert workflow is usability. Providers prefer to receive external alerts and view external data automatically within their EHR interface. Donohue et al. noted that HIE adoption would be facilitated through “reminders about external data, data displays combining both internal and external data, ease to extract and send data, clinical reports, and images to another provider and, conversely to incorporate external data into one’s own EHR”.^9^ This workflow requires VA RHIO team members to access a stand-alone Bronx RHIO alert report and manually enter alerts in an EHR template for providers to view the results. Providers that wish to obtain non-VA COVID-19 clinical information beyond test results must log onto the Bronx RHIO’s external website. A limitation of this study was that qualitative data and data on variations of the process that would indicate what worked better and for whom were not collected.

Another limitation of this workflow process is New York State consent requirements for HIEs. Consent is currently temporarily waived by the state during the pandemic for the VA. The VA previously also required opt-in consent for HIE use but shifted to an opt-out approach since April 2020 due to the Mission Act.^10^

A last limitation to this study is missing COVID-19 data from RHIO member partners, local non-member medical facilities, and missing COVID-19 deaths not reported to the JJP VAMC or RHIO members.

The VA is working toward greater interoperability with its community partners under VHA Office of Interoperability through the Veterans Health Information Exchange (VHIE) program.^9^ Through VHIE, the VA is currently connected to 135 eHealth Exchange members and 483 Direct Secure Messaging partners. VA providers access VHIE external data through a link from the VA EHR, and private sector data are viewable as HL7 C-CDA structured documents.^9^ As the VA transitions to the Cerner system, our experience is an instructive example of how HIE between VHA and community partners can improve service to Veterans.

## CONCLUSION

Health information exchange alerts can be used to identify facility patients diagnosed in the community for COVID-19 including those deceased and to facilitate ambulatory COVID-19 symptom follow-up of those patients by primary care teams at the VA. This study presents an example of a workflow which could be replicated by VA or non-VA sites to utilize HIE to link patients diagnosed with COVID-19 outside their primary source of health care to their primary care teams at their home facility for follow-up upon diagnosis or inpatient discharge until resolution of their infection.

## DATA AVAILABILITY STATEMENT

The data underlying this article cannot be shared publicly to protect the privacy of individuals included in the study.

## AUTHOR CONTRIBUTIONS

RS, KJ, NK, SR, JH, and KB designed the process and study. KJ, JH, SR, and RS curated the data. KJ and RS performed analyses. RS drafted the manuscript, and all authors contributed critical edits and approved the final manuscript.
